# Long-term snow-track indices of a Finnish native mesopredator declined while those of an invasive one increased

**DOI:** 10.1038/s41598-024-77777-w

**Published:** 2024-10-31

**Authors:** Vesa Selonen, Pyry Toivonen, Andreas Lindén

**Affiliations:** 1 Department of Biology, University of Turku, Yliopistonmäki, Vesilinnantie 5, Turku, FI-20014 Finland; 2 Natural Resources Institute Finland, Helsinki, FI-00790 Finland

**Keywords:** Climate change, Invasive animals, Invasive species, Mesopredators, *Nyctereutes procyonoides*, Snow-tracks, *Vulpes vulpes*, Boreal ecology, Invasive species

## Abstract

Monitoring both native and invasive species is crucial for understanding their ecological impacts. However, obtaining reliable data can be challenging, especially for elusive species like mesopredators. This study utilized snow-track surveys in Finland (1989–2022) to examine population trends of the invasive raccoon dog and the native red fox. While raccoon dogs are known to reduce activity during cold weather, we demonstrated that accounting for temperature and snowfall allows for effective population trend estimation using snow-track data. Track accumulation decreased in cold and snowy weather more clearly for raccoon dogs than for red foxes. We also found that the track accumulation of the raccoon dog had significantly increased, while those of the red fox population had declined, particularly in southern parts of the country. Notably, raccoon dog snow-track numbers increased in northern regions, suggesting a potential for further range expansion under a warming climate. These findings reveal a concerning shift in Finland’s mesopredators abundance due to the invasive species’ success and the decline of the native species. Thus, the invasive raccoon dog is likely to have an increasing role in those northern ecosystems where it interacts with the native fauna.

## Introduction

Estimating population abundances and trends is a crucial instrument in wildlife management and forms the basis for many management decisions^1,2^. These measures are needed to assess a population’s status and for choosing appropriate measures to support a healthy ecosystem and mitigate the declining of populations. On the other hand, data on population estimates can reveal the need for pest or predator control measures. Especially when dealing with invasive species with the potential to negatively affect or compete with local native species^3^.

Reliable estimation of occurrence patterns, such as population trends of a species, may not be a straightforward task. For example, the monitoring of mammalian mesopredators is complicated due to their nocturnal and secretive living habits^4,5^. This is the case for the raccoon dog (*Nyctereutes procyonoides*), which is the most common invasive mesopredator in large parts of Europe^3,6^. Raccoon dogs were introduced to the former Western Soviet Union for fur production from their original range in East Asia from. Since then, raccoon dogs have been called one of the most successful alien carnivores introduced to Europe^6^. The raccoon dog is an important predator of ground nesting birds and amphibians^7–9^. The expansion of raccoon dogs is also problematic from the perspective of the spread of zoonotic diseases and parasites^6,10^. Climate change is likely to further facilitate the spread of this species, as harsh winters are assumed to be a limiting factor to their habitable area^11^.

Mesopredator species ecologically comparable to the raccoon dog in northern and central Europe are the red fox (*Vulpes vulpes*) and the European badger (*Meles meles*)^6^. These three species, together with smaller carnivores such as the pine marten (*Martes martes*) form the mesopredator community in northern Europe. There are not necessarily antagonistic interactions between these species^12^, but through interspecific competition, the presence of the newcomer has the potential to negatively affect the native populations. Certainly, the invasive raccoon dog adds to the exploitation of common food sources. Assessments of raccoon dog population numbers have previously included surveys of hunting bag and roadkill numbers^6^. However, these methods often fail to provide an accurate view of population trends or distribution. Instead, snow-track surveys are commonly used to estimate indices of predator abundances^1,13–16^. Snow-track data can provide credible approximations of animal abundance and are useful for inferring population dynamics of species^13,17,18^, but see^14^ for a contrary discussion. However, it is important to acknowledge that such data are affected by both species’ density and activity.

The index for red fox abundance is assessed annually in Finland with snow-track data collected in the wildlife triangle scheme (Natural Resources Institute Finland, Luke^19^). This snow-track survey scheme includes also tracks of badgers and raccoon dogs, but for the badger snow-track surveys are a poor method for indexing badger populations because they mostly sleep during the boreal winter^20^. The raccoon dog’s wintering strategy includes alternating periods of physical activity and passivity^10,21^. That is, it can exhibit passive overwintering by winter sleep, which will decrease the snow-track accumulation. However, the species maintain some degree of winter activity even in very cold weather^10,22^. This suggests that snow-track data may be used to analyze occurrence of raccoon dogs, as long as weather variables are controlled for.

In this study, we utilized data from two type of annual snow-track surveys from the Finnish wildlife triangle scheme: (1) from surveys in landscapes of mix of agricultural and forested areas, hereafter referred to as “field triangles” and (2) from surveys in purely forest dominated landscapes, hereafter referred to as “forest triangles”. We, first, estimate the population trends for the invasive raccoon dog and the native red fox, and compare the results. We then predicted that: (i) snow-track numbers would remain lower for raccoon dogs compared to red foxes, especially under cold and snowy conditions, due to the raccoon dog’s reduced winter activity^22^; (ii) track numbers for both raccoon dogs and red foxes would be higher in field triangles, as these areas are located in more productive land than forested areas; and (iii) snow-track indices for both species would decrease in colder climates (i.e., in northern regions), but especially so for the raccoon dog which is abundant in southern Finland^23^. Additionally, we expect that warmer temperatures will more significantly increase track numbers in the northern parts of the study area compared to the south. The harsh climate in the north likely limits the activity of these species, particularly raccoon dogs, while higher temperatures may facilitate increased activity in these regions.

## Results

**Fig. 1:**
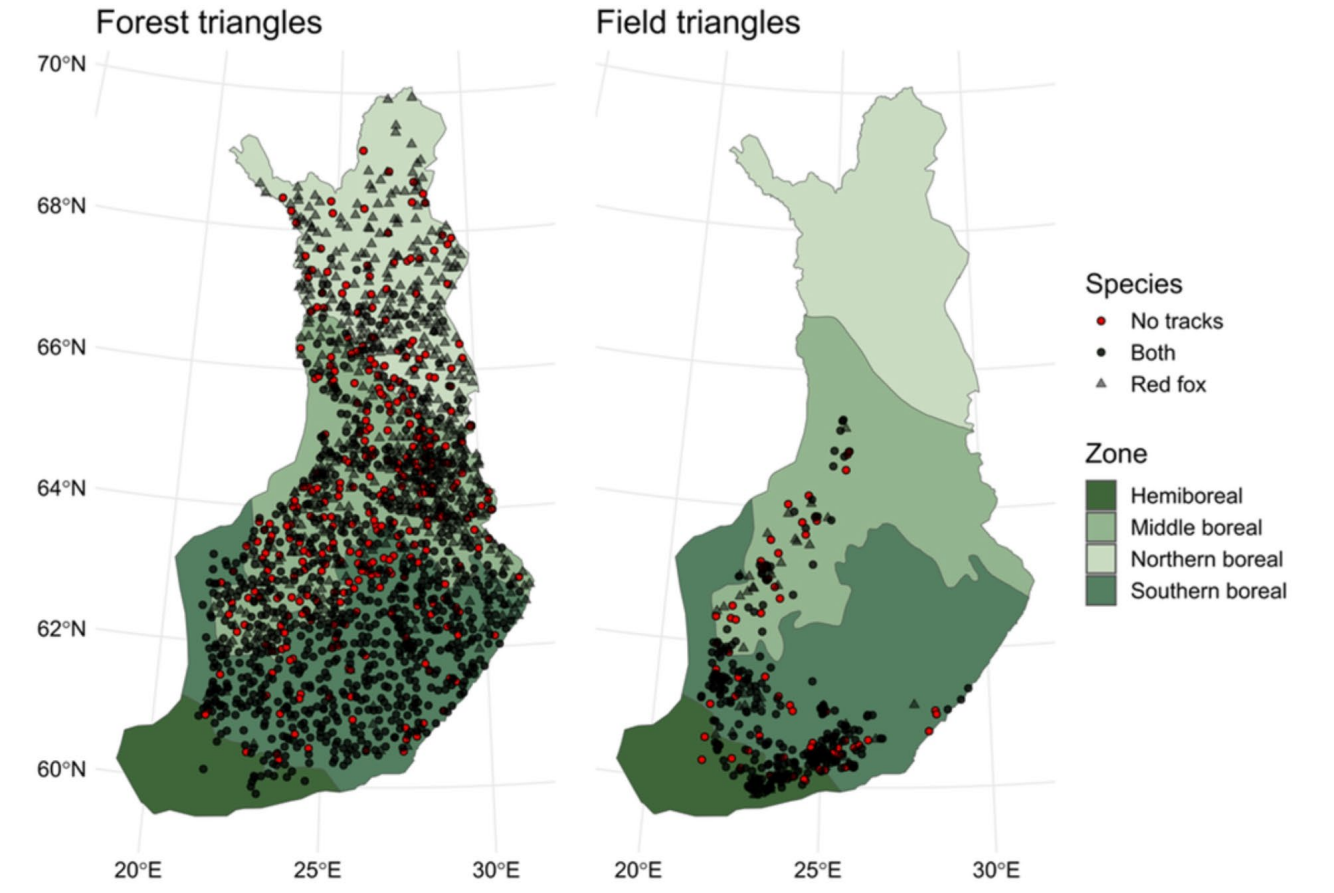
The distribution of the surveyed snow-track data. The black circles are sites with data for both species and triangles include data only for the red fox (all, except one site, reported tracks of red fox at least once). The cases without snow tracks are in red circles, which, in practice, are sites without data for raccoon dog. The left panel show the sites for forest triangles and the right panel shows the sites for field triangles.

Red fox produced tracks over the whole country but raccoon dog tracks were not observed in the northern parts of study area above 67.5°N (Fig. 1). The total count of snow-tracks was 48,980 for the red fox (*n* = 3,509 triangle surveys; i.e., combinations of triangle ID and year) and 5,068 for the raccoon dog (*n* = 3,064 triangle surveys) for the field triangle data. For the forest triangle data, the count of snow-tracks was 173,446 for red fox (*n* = 23,455 triangle surveys) and 8,272 for raccoon dog (*n* = 14,068 triangle surveys).

### Temporal trends

**Fig. 2:**
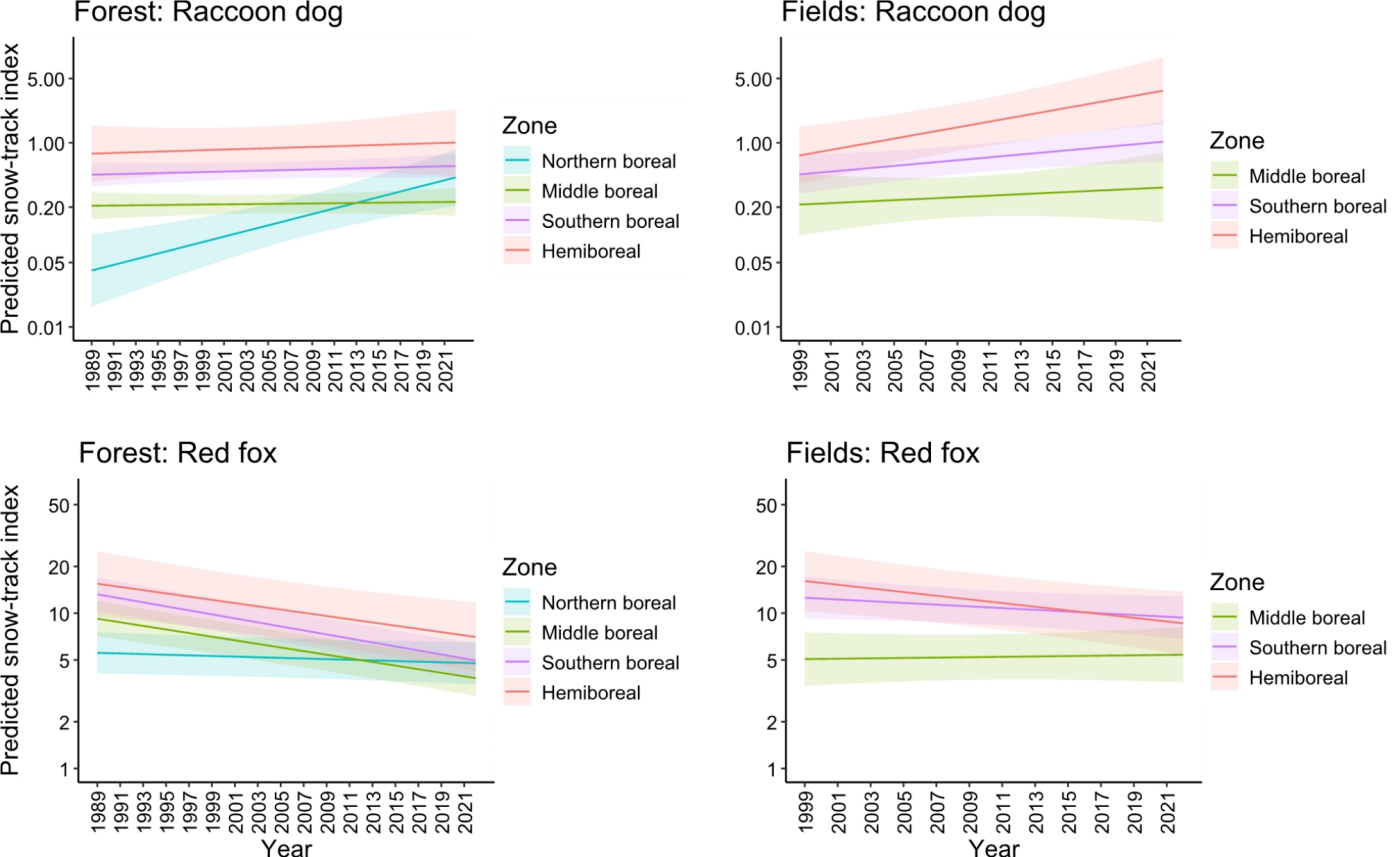
Raccoon dog and red fox predicted snow-track index (tracks / 10 km / day; shadowed areas are 95% confidence intervals) over time in forest and field triangles in different boreal zones in Finland.

The snow-track indices of the raccoon dog increased over time in field triangles (on average 3.8% / year; Table 1). In forest triangles, a clear increase was recorded only in the northern boreal zone (change 6.9% / year; Table 3; Fig. 2), the average change over all zones being 2.0% / year.

**Table 1:** Fixed and random effects parameters of model explaining raccoon dog snow tracks in field triangles (*n* = 3,066 surveys; 37.5% deviance explained).

Parameter	SD	Estimate ± SE	z-value	*P*
Intercept (South boreal)	0.43	–0.482 ± 0.193	–2.50	**0.01**
Temperature	0.50	0.635 ± 0.069	9.20	**< 0.001**
Snow depth	0.22	–0.295 ± 0.089	–3.29	**< 0.001**
Year.c	0.26	0.035 ± 0.015	2.31	**0.02**
Zone (Hemiboreal)		0.696 ± 0.264	2.64	**0.008**
Zone (Middle boreal)		–0.864 ± 0.301	–2.87	**0.004**
Temperature * Zone (Hemiboreal)		–0.214 ± 0.126	–1.75	0.08
Temperature * Zone (Middle boreal)		–0.411 ± 0.189	–2.18	**0.03**
Year.c * Zone (Hemiboreal)		0.035 ± 0.020	1.74	0.08
Year.c * Zone (Middle boreal)		–0.017 ± 0.027	–0.62	0.53
Random effect: Triangle ID	0.71			
Random effect: Association ID	0.42			
Random effect: Region ID	0.15			
Random effect: Year.f	0.42			

Signifiant values are in bold.

The fixed effects included weather effects and temporal trends in three boreal vegetation zones, ranging from the hemiboreal zone in the south to the middle boreal zone in the north, as well as the interaction terms Year. c*Zone and Temperature*Zone (see Fig. 1). The variation explained by the fixed effects (including all interactions with Zone) and the estimated variation of random effects on the intercept are reported as standard deviations (SD).

Year.c: year as continuous variable; Year.f: year as factor variable.

The snow-track indices of the red fox decreased over time in forest triangles in all vegetation zones except for the northern boreal zone (Table 4; Fig. 2), the average change over all zones being −2.2% / year. In field triangles, the red fox snow-track indices decreased in the hemiboreal zone (change −2.7% / year; Table 2; Fig. 2), the overall change being −1.3% / year.

### Track numbers

**Fig. 3:**
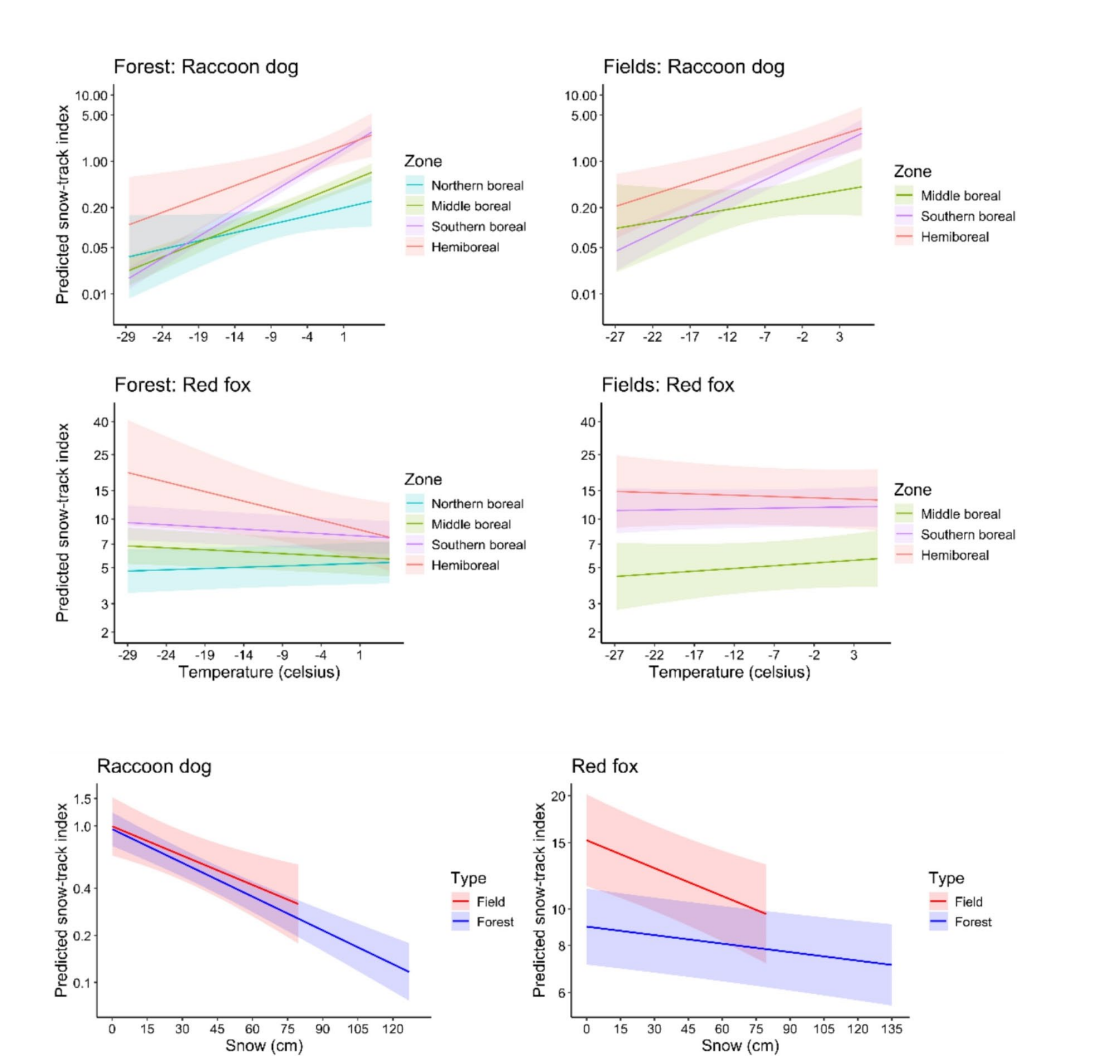
Effects of temperature (upper two panels, shown for each boreal zone) and snow depth (lowest panel) on raccoon dog and red fox snow-track indices in forest and field triangles, illustrated as predicted snow-track indices (tracks / 10 km / day; shadowed areas are 95% confidence intervals). The prediction for snow is shown for the Southern Boreal zone (snow had nonsignificant interaction with zone, that is, zones have identical slopes for the prediction).

Field triangles produced more tracks of red foxes than forest triangles, but for raccoon dogs the difference was not very clear (Figs. 2 and 3). Both the field triangles and forest triangles had more red fox snow tracks than raccoon dog snow tracks (Figs. 2 and 3). Overall model predicted average (± SD) track numbers in field triangles were 1.6 ± 2.3 and 14.6 ± 11.8, and in forest triangles were 0.6 ± 1.1 and 8.0 ± 7.7 tracks / day / 10 km, for the raccoon dog and the red fox, respectively. Snow-track indices decreased towards the north for both species (Tables 1, 2, 3 and 4).

**Table 2:** Fixed and random effects parameters of model explaining red fox snow tracks in field triangles (*n* = 3,512 surveys; 49.8% deviance explained).

Parameter	SD	Estimate ± SE	z-value	*P*
Intercept (South boreal)	0.32	2.531 ± 0.136	18.57	**< 0.001**
Temperature	0.02	0.009 ± 0.022	0.41	0.68
Snow depth	0.09	–0.133 ± 0.037	–3.55	**< 0.001**
Year.c	0.10	–0.013 ± 0.008	–1.54	0.12
Zone (Hemiboreal)		0.164 ± 0.164	0.99	0.32
Zone (Middle boreal)		–0.819 ± 0.194	–4.21	**< 0.001**
Temperature * Zone (Hemiboreal)		–0.0281 ± 0.0434	–0.64	0.51
Temperature * Zone (Middle boreal)		0.031 ± 0.046	0.65	0.51
Year.c * Zone (Hemiboreal)		–0.014 ± 0.007	–2.05	**0.04**
Year.c * Zone (Middle boreal)		0.015 ± 0.007	2.20	**0.03**
Random effect: Triangle ID	0.59			
Random effect: Association ID	0.22			
Random effect: Region ID	0.20			
Random effect: Year.f	0.26			

Significant values are in bold.

The fixed effects included weather effects and temporal trends in three boreal vegetation zones, ranging from the hemiboreal zone in the south to the middle boreal zone in the north, as well as the interaction terms Year. c*Zone and Temperature*Zone. The variation explained by the fixed effects (including all interactions with Zone) and the estimated variation of random effects on the intercept are reported as standard deviations (SD).

Year.c: year as continuous variable; Year.f: year as factor variable.

**Table 3:** Fixed and random effects parameters of model explaining raccoon dog snow tracks in forest triangles (*n* = 14,121 surveys; 35.8% deviance explained).

Parameter	SD	Estimate ± SE	z-value	*P*
Intercept (South boreal)	0.47	–1.188 ± 0.104	–11.46	**< 0.001**
Temperature	0.68	0.774 ± 0.036	21.67	**< 0.001**
Snow depth	0.34	–0.340 ± 0.042	–8.01	**< 0.001**
Year.c	0.16	0.007 ± 0.007	1.01	0.32
Temperature * Zone (Hemiboreal)		–0.299 ± 0.167	–1.79	0.07
Temperature * Zone (Middle boreal)		–0.254 ± 0.061	–4.15	**< 0.001**
Temperature * Zone (Northern boreal)		–0.479 ± 0.164	–2.93	**0.003**
Zone (Hemiboreal)		0.556 ± 0.273	2.04	**0.04**
Zone (Middle boreal)		–0.838 ± 0.111	–7.54	**< 0.001**
Zone (Northern boreal)		–1.347 ± 0.267	–5.05	**< 0.001**
Year.c * Zone (Hemiboreal)		0.002 ± 0.016	0.11	0.91
Year.c * Zone (Middle boreal)		–0.004 ± 0.006	–0.63	0.52
Year.c * Zone (Northern boreal)		0.064 ± 0.019	3.43	**< 0.001**
Random effect: Triangle ID	0.56			
Random effect: Association ID	0.25			
Random effect: Region ID	0.25			
Random effect: Year.f	0.33			

Significant values are in bold.

The fixed effects included weather effects and temporal trends in four boreal vegetation zones, ranging from the hemiboreal zone in the south to the northern boreal zone in the north, as well as the interaction terms Year.c*Zone and Temperature*Zone. The variation explained by the fixed effects (including all interactions with Zone) and the estimated variation of random effects on the intercept are reported as standard deviations (SD).

Year.c: year as continuous variable; Year.f: year as factor variable.

**Table 4:** Fixed and random effects parameters of model explaining red fox snow tracks in forest triangles (*n* = 23,542 surveys; 41.5% deviance explained).

Parameter	SD	Estimate ± SE	z-value	*P*
Intercept (South boreal)	0.19	1.621 ± 0.117	13.84	**< 0.001**
Temperature	0.03	–0.032 ± 0.010	–3.13	**0.002**
Snow depth	0.04	–0.041 ± 0.011	–3.45	**< 0.001**
Year.c	0.24	–0.030 ± 0.003	–9.81	**< 0.001**
Zone (Hemiboreal)		0.249 ± 0.191	1.30	0.19
Zone (Middle boreal)		–0.314 ± 0.077	–4.08	**< 0.001**
Zone (Northern boreal)		–0.472 ± 0.116	–4.06	**< 0.001**
Temperature * Zone (Hemiboreal)		–0.109 ± 0.068	–1.61	0.11
Temperature * Zone (Middle boreal)		0.004 ± 0.014	0.29	0.77
Temperature * Zone (Northern boreal)		0.051 ± 0.017	2.99	**0.003**
Year.c * Zone (Hemiboreal)		0.006 ± 0.007	0.81	0.42
Year.c * Zone (Middle boreal)		0.003 ± 0.002	1.89	0.06
Year.c * Zone (Northern boreal)		0.025 ± 0.002	12.41	**< 0.001**
Random effect: Triangle ID	0.48			
Random effect: Association ID	0.28			
Random effect: Region ID	0.41			
Random effect: Year.f	0.16			

Significant values are in bold.

These included weather effects and temporal trends in four boreal vegetation zones, ranging from the hemiboreal zone in the south to the northern boreal zone in the north, as well as the interaction terms Year.c*Zone and Temperature*Zone. The variation explained by the fixed effects (including all interactions with Zone) and the estimated variation of random effects on the intercept are reported as standard deviations (SD).

Year.c: year as continuous variable; Year.f: year as factor variable.

## Responses to weather variables

The snow-track indices for the raccoon dog decreased considerably with colder temperature and more snow in both data sets (Tables 1 and 3). In both data sets, the track index became low when there were > 50 cm snow and temperatures −10°C or colder (Fig. 3). Overall, for raccoon dog, temperature was a more powerful predictor than snow depth, although both contributed to explaining variation in the snow-track indices.

For the red fox, no effect of temperature was detected for field triangles, while snow-track indices slightly increased in forest triangles during colder weather in southern parts of the country (Tables 2 and 4; Fig. 3), contrasting with the strong effects of colder temperatures observed in raccoon dog snow track indices. Similarly to raccoon dogs, deeper snow decreased snow-track indices in red fox (Tables 2 and 4; Fig. 3). The negative effect of snow depth did not differ between the species in field triangles (difference in slopes = −0.13, SE = 0.091, *Z* = −1.41, *p* = 0.15), but was clearer for raccoon dogs than red foxes in forest triangles (difference in slopes = −0.30, SE = 0.038, Z = −8.17, *p* < 0.001).

We did not observe the predicted stronger effect of weather variables in the north than in the south. On the contrary, the result was the opposite for the raccoon dog as the effect of temperature on snow-track numbers was weaker in north than in south (interaction Temperature*Zone in Tables 1 and 3; Fig. 3).

## Discussion

Based on long-term snow-track surveys of Finnish mesopredators, we observed a declining trend for the native red fox, and an increasing trend for the invasive raccoon dog. The temporal trends, however, varied across the country. The raccoon dog was absent from northern parts of the study area in the subarctic region, but showed an increasing population trend near those areas. Supporting our predictions, there were fewer tracks in forest triangles than in field triangles and the track numbers declined in the north. The raccoon dog also produced clearly fewer snow-tracks than the red fox, as we expected. The raccoon dog produced clearly fewer snow tracks in cold and snowy weather, but still produced enough snow-track data to analyze temporal trends and provide proxies for regional abundance between vegetation zones. Contrary to our prediction, the response to weather variables were not stronger in harsh northern climate than in milder southern parts of the study.

## Temporal trends

Our results indicated that the invasive raccoon dog population is still expanding, especially in the northern boreal zone near the species’ invasion front. This is despite strong control measures (hunting) of raccoon dogs in these areas, as Finnish and Swedish wildlife managers aim to prevent the expansion to the north and west^24^. Currently, the raccoon dog shows a clearly more southern distribution pattern in Finland compared to the red fox, but nevertheless, it appears to be able to live in very cold environments^21,22^. Thus, it can be expected to expand further north as the climate warms.

In addition to the increase in raccoon dog track numbers, our results imply that the native red fox is declining in Finland. The reason for this decline remains unclear, but also some other common native mammals show declining trends in forest landscapes in Finland. For example, Turkia et al.^25^, observed red squirrel, *Sciurus vulgaris* (potential prey for red foxes), declines over large areas in boreal forests. They linked the decline to warming climate, although increased forest management may also play a role. Regarding our data, the declining trend of the red fox appeared clearer in forest triangles than in field triangles. The field triangles were in landscapes with a mixture of agriculture and forests, and those areas might provide more stable food resources for red foxes than pure forests^17^. Changes in prey species communities are expected to affect red fox population dynamics and might thus be related to declining population trends^26,27^. For example, voles are important prey for red foxes and their abundances fluctuate cyclically. There were irregularities in these cycles in the 1990s, but the cyclicity may have partially returned since then^28^. Apex predators, such as wolf, *Canis lupus*, kill mesopredators and have increased in Finland in recent decades, but previous analyses indicate that raccoon dogs may be more negatively affected by apex predator presence compared to red foxes^29^, see also^30^. Red foxes and raccoon dogs both benefit from carrion produced by apex predators^31^; for a review on the factors affecting red fox abundance see e.g^32^. In general, in Europe, the red fox increased after rabies control in the 1980s but also due to changes in landscape structure^33,34^. After that, the red fox populations in western and central Europe have been mainly stable or slightly increased^34,35^ (see also e.g^36^). although opposite examples also exist^34^. In Scandinavia, the red fox population increased in the 20th century^31^, but results for recent decades are less clear, but see^36^. In hunting statistics of Finland, the number of red foxes killed has been declining from 50,000 to 60,000 to 40,000–50,000 individuals killed yearly during 1996–2022. Instead, during the same time period raccoon dog hunting bag has been about double that of red fox, increasing from 50,000 to 100,000 to about 150,000 killed individuals yearly (data by Natural Resources Institute Finland). That is, the hunting pressure does not seem to explain the trends observed in the current study. Instead, they seem to show the same trend as we observed.

The red fox decline was clearer in the south than in the north. The reason for this pattern remains unknown. The density of the raccoon dog is high in the southern Finland^23^, but the possibility for competition between these two species remains unclear. Drygala and Zoller^37^ concluded that differences in space use likely prevent competition between red foxes and raccoon dogs in the agricultural landscape of northeast Germany. Food niche overlap studies suggest also that these mesopredators can coexist^12,38^ and Kowalczyk and Zalewski^39^ concluded that there is no evidence for nest competition. However, both species do utilize similar sites for nesting^6^, which could create the possibility for nest competition if nest sites are scarce. Usage of the same nest sites also increases the transmission of diseases from raccoon dogs to red foxes. The population size of the latter is known to potentially be affected by sarcoptic mange^26,40^, which is spread by raccoon dogs. A simulation study indicated that raccoon dogs, when reaching high density, could outcompete native red foxes^41^. Additionally, Kauhala^42^ claimed that the red fox population started to increase when that of the raccoon dog decreased due to intensive hunting in a study area in Finland. Thus, more research on the interspecific interaction between these mesopredators, an invasive and a native one, would be needed. In particular, this may be relevant in areas where the density of the raccoon dog is already above that of the red fox^23^.

## Track numbers

The forest triangles had lower snow-track indices of red foxes compared to field triangles. This is according to our prediction and likely related to the fact that forest-dominated landscapes are less productive than agriculture-dominated landscapes. For the raccoon dog, the difference in track numbers between forest and field triangles was surprisingly small, although it is known that the species does not prefer purely forest-dominated areas^43^. In general, the track numbers were at quite low level for the raccoon dog and tracks were not observed in the northern parts of the study area (forest triangles; Fig. 1). It is known that raccoon dogs are sometimes found in these areas and are active in winter^22^. The species just remains so rare that no snow tracks were detected. The observed temporal trends remained also statistically quite weak for the raccoon dog. The changes in predicted track indices indicated clear changes in time, but there was high variation in the data, which is related to the low number of tracks for raccoon dogs.

In all parts of the country the raccoon dog produced less snow tracks than the red fox, despite the fact that the raccoon dog is the most abundant mesopredator in the southern parts of Finland^23^. It is concluded that for estimating carnivore abundances snow tracking is the most efficient approach to detect winter-active species^44^, but it is important to acknowledge that such data hold information about both species’ density and activity. In our case, the track numbers do not reliably reflect average differences in the abundance of the studied species, but are much determined by the lowered winter activity of the raccoon dog. Even in moderate winter conditions, we suggest that the production of snow tracks in raccoon dogs remains at a lower level than in species that are active the whole winter, such as the red fox. It should be also noted that the data we used are citizen science data, although produced by trained volunteers. The raccoon dog inhabited the whole country, including the northern parts, already in 1950s^6^. That is, before the start of the used snow-track survey scheme, the raccoon dog had been present in the whole study area for over 40 years. Thus, the species is well known by the persons doing the surveys thorough the country and it seems unlikely that there would be relevant biases in reporting that would affect the observed trends in this study. The snow tracks of these two species are also relatively easy to separate from each other.

### Responses to weather

The climate is expected to be the main factor controlling the raccoon dog expansion^11,45^. However, based on current and earlier results^21,46,47^ the species is active in quite cold weather. Its activity clearly decreases during cold nights and in mid-winter, and, in comparison, the red fox was not affected by cold temperature in our study. Similar to raccoon dogs, red fox snow-track density declined when snow depth increased, although the response was stronger in the raccoon dog. Likely, both movement and foraging become harder in deep snow^48^, decreasing snow-track accumulation. For the raccoon dog it is earlier concluded that it stays increasingly tied to the nests in winter when the temperature drops below −10℃ and the snow depth is more than 35 cm^46^. Our results support this conclusion. In any case, it is clear that the species can thrive in very snowy conditions both in the invaded range and in the species native range in southern Siberia^6^.

We predicted that the effect of weather variables would be stronger in the north than in the south. The rationale here would be that because the cold temperature and deep snow likely limit the species, this would be particularly a problem in north in harsh environment. Thus, variation in these variables would have particularly strong effect in north. However, we did not find this and actually the opposite was the case for raccoon dogs. Apparently, the winters in north remain so cold that variation in temperature less affects winter activity of the species than it affects in south. Although the climate change has warmed winter weather especially in northern Finland during recent decades^49^, this has not changed the fact that the winter in northern Finland still remains severe. Instead in south, the mild winter weather often means low amount of snow and plus degrees, which seldom is the case in northern Finland. Thus, raccoon dogs apparently increase more their activity levels in warm winter weather in south than they do in the northern Finland.

## Conclusions

We conclude that snow-tracks surveys are useful in the monitoring of the invasive raccoon dog, but track accumulation strongly decreases in colder weather and deeper snow, calling for compulsory inclusion of weather covariates when studying raccoon dog snow tracks. The red fox shows weaker effects of weather, and temperature has no effect or has the opposite effect (moves more in cold conditions) compared to raccoon dog. Our results also indicate that the mesopredator community in boreal Finland has changed due to the increasing population of the invasive species and the decreasing population of the native mesopredator, the red fox. Climate change is projected to become the primary driver of biodiversity change in northern ecosystems^50–53^, which is likely to further benefit the raccoon dog. Thus, the invasive raccoon dog is likely to have an increasing role in boreal and (sub)arctic ecosystems where it interacts with the native fauna.

## Materials and methods

### Study species and study areas

The raccoon dog is an omnivorous canid predator that weighs typically 5 kg in early summer and about 9 kg in autumn before winter sleep^54^. It is monogamous, dens in pairs and can produce litters of up to nine offspring each year, starting at the age of one year. Raccoon dogs invaded Finland from the Soviet Union in the 1950s, and its current range covers most of the country except for the northern parts of Lapland^6^. The raccoon dog’s European range is expanding, mainly consisting of Eastern and Northern Europe, Germany and Denmark^6^. In southern Finland, the raccoon dog may currently be the most common mesopredator^23^, but it has a more southern distribution in Finland than red foxes have^6^.

The red fox weighs typically 5–8 kg. It dens solitarily, in pairs, or in family groups, depending on food resources^55^. While also an opportunistic omnivore, the red fox tends to be more predatory than the raccoon dog, feeding more frequently on birds and small mammals^56^. Reproduction in red foxes starts at the age of one year, when they produce litters of five offspring on average^20^. The species is active during the whole winter, but the levels of activity decrease during the cold months^20^. The red fox is found throughout Finland from the hemi- and southern boreal zones of southern Finland to the northern boreal or subarctic regions in Lapland.

This study was conducted in the boreal forest-dominated landscape of Finland (Fig. 1). The main tree species in these mostly managed forests are Scots pine (*Pinus sylvestris*), Norway spruce (*Picea abies*), and birches (*Betula* sp.). Bodies of water, agricultural areas, and bogs fragment the forest landscape. Large bog areas are found mainly in the northern parts of the country. Agricultural areas and urban areas are found more in southwestern and southern Finland, but occur sparsely throughout the whole country.

### Snow-track data

Snow-track data contains two types of fixed survey routes, which are shaped like equilateral triangles: (i) 3 × 4 km wildlife triangle routes in forest habitat, i.e. the “forest triangles”; and (ii) 3 × 2 km triangle routes in a mosaic-like landscape of agricultural land mixed with forests, mainly distributed along the coast, i.e. the “field triangles”^57^. Forest triangles were surveyed annually during 15th January–29th February (in north − 15th March) and field triangles 1st January–29th February. Triangle surveys have been performed in Finland each winter since 1989 for forest triangles and since 1999 for field triangles. The snow tracks of the two studied species are relatively easy to differentiate and tracking data were collected by volunteers (typically local hunters) after they were trained in track identification. Thus, misidentification was likely a problem of minimal concern but we do not have any data to confirm or dispute this. On average, the citizen-science data used included 730 forest triangles and 170 field triangles surveyed each year.

The number of animal tracks observed is typically related to the transect length surveyed and the number of days (24 h) of snow-track accumulation and is hence reported as a snow-track index (i.e., a density with the unit: tracks / 10 km / day). We used data from 1999 to 2022 for field triangles and from 1989 to 2022 for forest triangles. The snow-track survey is conducted once a year in each triangle, when possible, but very few triangles have been surveyed every year. Some triangles have been founded after the start of the monitoring scheme, some have become inactive, and there are occasionally missing years in the middle. We only included triangle routes that had data from at least 5 years and had at least one year of observations of the species of interest. After this the length of time series varied between 5 and 34 years (average 16.1, median 15) for forest triangles and 5 and 22 years (average 10.9, median 10) for field triangles.

Weather data were extracted using coordinates of each triangle from nearest interpolated grid cells (grid size 1 × 1 km^2^) produced by the Finnish Meteorological Institute (FMI). We extracted daily data of snow depth (cm) and temperature (℃) for each survey year and then calculated the mean values from the periods of track accumulation for each survey. These periods were the time-interval between the survey (that lasted few hours) and last snowfall before the survey (covering the old tracks). We filtered data to only include the data points where the track accumulation period was at maximum 7 days (the average accumulation period length in the used data was 1.6 ± 0.9 (SD) days).

Weather data extraction from grid cells was done using the ncdf4-package v. 1.21^58^ in R v. 4.2.0^59^.

### Analysis

We modelled the snow-track index for field triangles and forest triangles using generalized linear mixed models, with negative binomial error distribution and a logarithmic link function. The models were fitted separately for red fox and raccoon dog, and for forest and field triangles, the dependent variable being the number of snow tracks per triangle per survey identified to originate from the focal species.

As an offset variable, we applied the natural logarithm of survey effort, which in turn is defined as “time for snow-track accumulation” (days) × “length of survey route” (10 km). Using this offset variable in a log link model, where the number of tracks is the response variable, is equivalent to a situation where the fixed effect (non-offset) variables describe changes in the snow-track index, with the unit: tracks / day / 10 km.

Independent variables included in the model were the average daily temperature (Celsius) and snow depth (cm) from the period of snow-track accumulation, year (continuous), and the boreal zone in which each triangle was situated (four-level factor variable, see Fig. 1). We included interaction terms between boreal zone and temperature in the models, but to simplify we left out the interaction between snow and boreal zone, because during initial model checking it was not significant in any of the modes. We also included the interaction of the boreal zone and year (as a continuous variable) in the models, to see whether there were differences between the temporal trends in different parts of the country. For each species, temperature and snow depth were scaled to zero mean and unit variance, for better model convergence properties. Also, continuous variable year was centered to zero mean for each species. Scaling the continuous variables over both forest- and field triangles enabled us to compare the snow-track indices under the same (average) conditions.

The data were spatially autocorrelated and to account for this in the unexplained variation of the model, we included the following factor variables: (1) wildlife agency region identity (largest spatial scale), (2) hunting association region identity (medium spatial scale) and (3) triangle identity (small spatial scale), as nested random effects on the intercept. The wildlife agency region divides Finland to 15 separate regions and the hunting association regions divide the country to 279 separate areas (https://riista.fi/en/game-administration/). In addition, survey year as a factor variable was included as a separate random effect to account for unexplained temporal variation. Together, these terms account for unexplained variation in time and space, which was not explained by the rough temporal trends, variation by vegetation zones or weather. With this setting, the Moran’s I for the extracted random effects of triangle identity was close to zero (between −0.02 and 0.02 analyzed with spdep-package v. 1.3-5^60^) indicating low level of remaining spatial autocorrelation in any of the models.

The statistical analyses were done using glmmTMB-package v. 1.1.9^61^ in R v. 4.4.1^59^.

## Data Availability

The datasets analyzed during the current study are owned by Natural Resources Institute Finland (Luke) and are not publicly available but are available from the corresponding author on reasonable request.
